# Resistant Starch from Purple Sweet Potatoes Alleviates Dextran Sulfate Sodium-Induced Colitis through Modulating the Homeostasis of the Gut Microbiota

**DOI:** 10.3390/foods13071028

**Published:** 2024-03-27

**Authors:** Zhihao Wang, Min Gao, Juan Kan, Qingyang Cheng, Xiaotong Chen, Chao Tang, Dan Chen, Shuai Zong, Changhai Jin

**Affiliations:** College of Food Science and Engineering, Yangzhou University, Yangzhou 225127, China; pf56cc@163.com (Z.W.); gaomin0727@163.com (M.G.); 15380819049@163.com (Q.C.); a185092117@163.com (X.C.); 008155@yzu.edu.cn (C.T.); dchen@yzu.edu.cn (D.C.); shuaizong@yzu.edu.cn (S.Z.); chjin@yzu.edu.cn (C.J.)

**Keywords:** purple sweet potato, resistant starch, gut microbiota

## Abstract

Ulcerative colitis (UC) is a complicated inflammatory disease with a continually growing incidence. In this study, resistant starch was obtained from purple sweet potato (PSPRS) by the enzymatic isolation method. Then, the structural properties of PSPRS and its protective function in dextran sulfate sodium (DSS)-induced colitis were investigated. The structural characterization results revealed that the crystallinity of PSPRS changed from C_A_-type to A-type, and the lamellar structure was totally destroyed during enzymatic hydrolysis. Compared to DSS-induced colitis mice, PSPRS administration significantly improved the pathological phenotype and colon inflammation in a dose-dependent manner. ELISA results indicated that DSS-induced colitis mice administered with PSPRS showed higher IL-10 and IgA levels but lower TNF-α, IL-1β, and IL-6 levels. Meanwhile, high doses (300 mg/kg) of PSPRS significantly increased the production of acetate, propionate, and butyrate. 16S rDNA high-throughput sequencing results showed that the ratio of *Firmicutes* to *Bacteroidetes* and the potential probiotic bacteria levels were notably increased in the PSPRS treatment group, such as *Lactobacillus*, *Alloprevotella*, *Lachnospiraceae*_NK4A136_group, and *Bifidobacterium*. Simultaneously, harmful bacteria like *Bacteroides*, *Staphylococcus*, and *Akkermansia* were significantly inhibited by the administration of a high dose of PSPRS (*p* < 0.05). Therefore, PSPRS has the potential to be a functional food for promoting intestinal health and alleviating UC.

## 1. Introduction

Inflammatory bowel disease (IBD) in humans is a chronic disease characterized by gastrointestinal inflammation, and its prevalence is increasing globally [1]. As a major manifestation of IBD, ulcerative colitis (UC) has symptoms such as intestinal mucosa damage, relapsing easily, abdominal pain, diarrhea, and bloody stools [2]. Thus far, the pathogenesis of UC is largely unclear; it may be affected by genetic factors, environmental factors, infection, gut microbiota, and immune factors [3,4]. When the human body suffers from UC, it will cause gut barrier dysfunction. According to research findings, the destruction of the intestinal barrier led to the transportation of bacterial lipopolysaccharide to the intestine, which resulted in promoting an abnormal immune response [5]. The complementary medical treatments for UC mainly include amino salicylic acids, immunosuppressants, corticosteroids, and biological agents [6]. However, several disadvantages have been reported as a consequence of those medical therapies, including high cost, adverse side effects, and increased drug resistance [7]. Thus, it is crucial to find alternative therapies with weaker side effects and higher efficacy for treating UC, particularly those with active ingredients from natural plants.

The human gut microbiota constitutes a complex and rich ecosystem harboring trillions of microorganisms. It is becoming increasingly evident that the intestinal microbiota is closely connected to gut movement, gut barrier function, and immune system maturation. Recent studies showed that gut microbiota dysbiosis is a trigger contributing to conditions of many diseases, such as obesity, type 2 diabetes, Crohn’s disease (CD), and UC [8,9,10]. The gut microbiome in patients with UC is primarily characterized by reduced microbial diversity and the expansion of *Proteobacteria* [11]. Fortunately, abnormal gut microbiota in patients with UC can be restored by metabolites, including short-chain fatty acids (SCFAs), which are produced by intestinal microbiota fermentation of non-digestible carbohydrates [12].

Sweet potato (*Ipomoea batatas* L.) is considered an important root crop globally because of its extensive adaptability, high yield, and rich nutrition [13]. Purple sweet potato (PSP) exhibits a dark purple color owing to its high content of acylated anthocyanins. Recent evidence suggests that purple sweet potatoes exhibit various biological effects, including anti-inflammatory, anti-tumor, anti-oxidant, and liver-protective [14,15,16,17]. However, previous studies had mainly focused on anthocyanins and polysaccharides, whereas the resistant starch (RS) in purple sweet potatoes had not been fully studied. Purple sweet potato is mainly used nowadays as a source of pigments and polysaccharides, whereas its rich starch stores are not being fully utilized, resulting in wastage of resources. Generally, purple sweet potato starch is easier to retrograde and form RS due to its higher amylose content [18]. RS is considered a component of dietary fiber that is not readily digested and absorbed in the small intestine and could be fermented in the colon by the intestinal microbiota with the production of SCFAs, especially butyric acid, which has been particularly identified for human health, including maintaining gut function and integrity, modulating immune and inflammatory responses, and lowering the risk of colon cancer [19]. Previous studies found resistant starch was beneficial to dinitrobenzene sulfonic acid (TNBS)-induced UC via prebiotic and butyrate effects [20]. In addition, RS had the advantages of safety, availability, and low cost. Research has not been conducted extensively on purple sweet potato RS function and structure. In this study, resistant starch was produced from purple sweet potato (PSPRS) by protease, α-amylase, and amyloglucosidase. Then, the structure of PSPRS was characterized, and the alleviating effect of PSPRS on DSS-induced colitis mice was evaluated to provide a certain basis for PSPRS as a functional additive to treat or prevent UC, with a view to extending the purple sweet potato RS industry chain and adding its value.

## 2. Materials and Methods

### 2.1. Experimental Materials

Purple sweet potatoes (Xuzi No. 8) were provided by the Xuzhou Institute of Agricultural Sciences (Xuzhou, China). Dextran sulfate sodium (DSS, purity of 98% and molecular weight of 40 kDa) was purchased from MP Biomedical Co. (Santa Ana, CA, USA). Alfa amylase from porcine pancreas, amyloglucosidase, and dimethylsulfoxide (DMSO) were purchased from Sigma-Aldrich Co. (St. Louis, MO, USA). All enzyme-linked immunosorbent assay (ELISA) kits were purchased from Nanjing SenBeiJia Biological Technology Co. (Nanjing, China). All other chemical reagents were analytical grade and purchased from Sinopharm Chemical Reagent Co., Ltd. (Shanghai, China).

### 2.2. Starch Isolation

The starch in purple sweet potato (PSPS) was prepared following the method described in Guo et al. [21]. In brief, the fresh purple sweet potatoes were blended in a juice extractor for 10 min with deionized water. Then, the homogenate was successively filtered and settled overnight. The precipitated starch was extracted 3 times with 95% ethanol for 8 h at 45 °C to remove most of the lipids, protein, pigments, and polysaccharides. After that, precipitated starch was treated with NaOH, deionized water, and anhydrous ethanol successively. Finally, the sample was dried at 45 °C for 24 h to obtain PSPS.

### 2.3. Preparation of Resistant Starch

The preparation method of purple sweet potato resistant starch (PSPRS) was according to Gani et al. [22], with slight modifications. A starch sample (1 g PSPS) was added to 5 mL of phosphate buffer (0.2 mol/L, pH 5.2) containing 5 mg of protease and then incubated at 37 °C for 1 h. The mixture was centrifuged at 3000× *g* for 10 min, then treated with α-amylase and *amyloglucosidase*, and incubated at 37 °C for 16 h. After incubation, the mixture was centrifuged at 5000× *g* for 15 min, and the residue was retained. The obtained residue was washed twice with anhydrous ethanol, followed by washing with ethanol, dried at 45 °C in a hot air dryer (ZRD-7140, Zhichen Instruments, Shanghai, China), and then ground to obtain PSPRS for further analyses.

### 2.4. Chemical Composition

The water content was determined by using a hot air dryer. The starch content was measured according to the method of Gosta et al. [23]. The protein and ash content were analyzed by the AOAC method 2001.11 (2005) and AOAC method 938.08 (2005), respectively [24]. The amylose content was analyzed following the procedure described by Wang et al. [13]. The resistant starch content was determined by the method of McCleary [25].

### 2.5. Physicochemical Properties

#### 2.5.1. Scanning Electron Microscopy (SEM)

The SEM of the samples was determined according to the method of Xie et al. [26]. The starch sample was adhered to the double-sided adhesive tape mounted on an aluminum stub and then sputter coated with gold under vacuum. The sample was observed with a S-4800 SEM (Hitachi Ltd., Tokyo, Japan) operated at 15 kV.

#### 2.5.2. Color of Starch

The colors of the samples were determined according to the method of Kan et al. [27]. The colors of PSPS and PSPRS were measured using a chromameter (Wave, WR-10, China). The chromameter was calibrated using a standardized white tile before measurement. L value, a value, and b value represented lightness, reddish-greenish, and yellowish-bluish, respectively.

#### 2.5.3. Water-Binding Capacity (WBC)

The WBC was determined according to the method of Xu et al. [28]. 0.3 g of dry starch was added in 40 mL of distilled water, followed by constant stirring for 1 h, and then centrifuged at 3000× *g* for 10 min. Excess water was removed from the settled starch layer, which was then continuously drained for 10 min at room temperature, and then wet starch was weighed. The formula for calculating WBC is as follows:

WBC (%) = (W_wet starch_ − W_dry starch_)/W_dry starch_ × 100%

#### 2.5.4. X-ray Powder Diffractometry (XRD)

The crystalline structures of PSPS and PSPRS were analyzed with an X-ray diffractometer (Bruker AXS GmbH, Karlsruhe, Germany) according to the method of Xie et al. [26]. The diffractometer was operated at a voltage of 40 kV and a current of 40 mA. The sample was scanned over the region of 3°–40°(2θ) with a step size of 0.02. The degree of crystallinity of the sample was analyzed using the ratio of the crystallinity area to the total diffraction area.

#### 2.5.5. Solid-State Cross-Polarization Magic Angle Spinning Nuclear Magnetic Resonance (^13^C CP/MAS NMR)

The solid-state NMR spectra of PSPS and PSPRS were recorded on a Bruker Avance III 400WB spectrometer (Bruker Biospin GmbH, Ettlingen, Germany) equipped with a double resonance H/X CP-MAS 4 mm probe according to the method of Zhang et al. [29]. The spectrum was obtained by using the standard CP/MAS technique with a spinning rate of 6 kHz and a magic angle of 54.7°. The cross-polarization contact time was 1.2 ms with a recycle delay of 3 s for an acquisition time of 15.7 ms.

#### 2.5.6. Small-Angle X-ray Scattering (SAXS)

SAXS analysis was performed according to the method of Feng et al. [30]. Briefly, the starch sample was dispersed in an excess of distilled water to form slurries and stored at 4 °C for 12 h. Then, the SAXS pattern was recorded on the Bruker NanoStar SAXS system (Bruker AXS GmbH, Karlsruhe, Germany) equipped with a Vantec 2000 area detector. The optics and sample chamber were under vacuum to minimize air scattering while the SAXS pattern was collected.

#### 2.5.7. Fourier-Transform Infrared Spectroscopy (FTIR)

The FTIR spectra of PSPS and PSPRS were analyzed on a Varian 670 FTIR spectrometer (Varian Inc., Palo Alto, CA, USA) according to the method of Xie et al. [26]. The samples were analyzed at a resolution of 4 cm^−1^ by 64 scans at room temperature. Spectra were baseline-corrected in the range of 1200–900 cm^−1^ before deconvolution was applied using Omnic version 8.0 software (Thermo Fisher Scientific Inc., Waltham, MA, USA). A half-band width of 19 cm^−1^ and a resolution enhancement factor of 1.9 with triangular apodization were employed. The absorbance height ratio of 1047 cm^−1^/1022 cm^−1^ was calculated from the deconvoluted spectrum.

### 2.6. Animals and Treatments

#### 2.6.1. Animal Experimental Design

Four-week-old male ICR mice were supplied by the Comparative Medical Center of Yangzhou University. All mice were fed at 23 °C with a 12 h light/dark cycle and were accessing freely standard chow (Xietong Pharmaceutical Bioengineering Co., Ltd., Nanjing, China) and water.

The experiments were carried out in accordance with the guidelines of the National Research Council’s Guide for the Care and Purpose of Laboratory Animals issued by the People’s Republic of China and approved by the Ethics Committee of Experimental Animal Care at Yangzhou University (license number SCXK2017-0007). Twenty-four mice were randomly split into four groups (n = 6 per group), including (a) the normal control group (NC); (a) the DSS group; (c) the DSS+ low dose of PSPRS group (DSS+LPSPRS); and (d) the DSS+ high dose of PSPRS group (DSS+HPSPRS). Each mouse was housed in an individual cage. The DSS group, DSS+LPSPRS group, and DSS+HPSPRS group were treated with 4.0% of DSS for 7 days, whereas drinking water was given to the control group. From day 1 to day 24, the DSS+LPSPRS group and DSS+HPSPRS group were given 100 mg/kg and 300 mg/kg of PSPRS, respectively, and saline was given to the other two groups by gavage once a day. During the fecal collection period, which occurred within 3 days prior to the mice being sacrificed, feces were subsequently collected from a stainless steel tray positioned beneath the cages for subsequent analysis.

Mice were weighed daily, and blood samples were taken from anesthetized animals by retroorbital puncture and then sacrificed by cervical dislocation. The spleen, thymus, and colon were then collected for further research.

#### 2.6.2. Immune Organ Index Analysis

The spleen and thymus indexes were calculated based on the following formula:

Spleen or thymus index = thymus or spleen weight (mg)/body weight (g)

#### 2.6.3. Histological Analysis

The histological analysis was determined according to the method of Kan et al. [31]. The distal colon tissues of the mice were collected and fixed in 4% paraformaldehyde for 24 h after removing the appendant tissues. Tissues were then dehydrated with gradient ethanol and embedded in paraffin, which were sliced into 3-µm sections. Hematoxylin and eosin (H&E) were used as dyes. Images were obtained by using a microscope. Tissues were sliced into 3 µm thick sections, and the sections were deparaffinized in xylene and dehydrated through graded ethanol, followed by staining using hematoxylin–eosin. Images were obtained by using a microscope. Images were obtained by using a Zeiss Axioskop microscope (Zeiss, Oberkochen, Germany).

#### 2.6.4. Measurement of Cytokines and IgA in the Colon

The levels of IL-6, IL-1β, IL-10, TNF-α, and IgA in the colon tissue of mice were determined by ELISA kits (Jiancheng Bioengineering Institute, Nanjing, China), in accordance with the manufacturer’s operating manuals. The absorbance was measured using a microplate reader (GO, Thermo Scientific, Waltham, MA, USA) at 450 nm.

### 2.7. Short-Chain Fatty Acid Analysis

The contents of SCFAs in the feces of mice, including acetate, propionate, and butyrate, were evaluated based on the method of Liu et al. [32]. The column was a DB-Wax capillary column (30 m × 0.25 mm). Prior to analysis, 100 mg of feces were added to a 2 mL centrifuge tube with 0.4 mL of distilled water and 0.1 mL of sulfuric acid (0.5 mol/L). After vibration and centrifugation at 10,000× *g* for 10 min, 0.5 mL of supernatant was mixed with 0.5 mL of diethyl ether and blended again. After centrifuging at 10,000× *g* for 10 min, the supernatant was injected into a gas chromatographic column. Quantitative analysis of acetate, propionate, and butyrate was calculated from the peak areas of the internal standard (caprylic acid methyl ester) using the TurboMass program (version 6.1.2).

### 2.8. High-Throughput 16S rRNA Sequencing

Total microbial DNA from mouse feces was extracted with the Stool DNA kit (Tiangen Biotech Co., Ltd., Beijing, China). Amplification of the V3–V4 region of the bacterial 16SrRNA was conducted mainly by PCR with the primers 338F (ACTCCTACGGGAGGCAGCAG) and 806R (GGACTACHVGGGTWTCTAAT). The PCR products were extracted using a QIAGEN Quick Gel Extraction Kit. For each sample, PCR amplicons were sequenced with the Illumina Miseq platform. The construction of related libraries and high-throughput sequencing were performed by Majorbio Bio-Pharm Technology Co., Ltd. (Shanghai, China).

### 2.9. Statistical Analysis

All data were shown as the mean ± standard deviation (SD). A one-way analysis of variance (ANOVA) followed by Tukey’s significant difference test was conducted using SPSS 17.0 software.

## 3. Results and Discussion

### 3.1. Physicochemical Properties

The physicochemical properties of PSPS and PSPRS are shown in Table 1. The protein contents of PSPS and PSPRS were lower than 1%, indicating that the purities of PSPS and PSPRS were relatively high [33]. The protein and starch contents of PSPRS showed a significant difference from PSPS (*p* < 0.05), indicating the destruction of structure upon enzymatic hydrolysis [34]. Compared with PSPS, the amylose content of PSPRS was significantly improved (*p* < 0.05). This may be attributed to the effect of amyloglucosidase and amylase on the α-(1-6) linkage of amylopectin. Similar findings were reported by Sang et al. [35]. The resistant starch (RS) content of PSPRS was notably increased from 28.12% (PSPS) to 71.64% due to the increased amylose content of PSPRS (*p* < 0.05). As mentioned in the literature, a positive correlation was observed between the RS content and the amylose content [36]. In addition to amylose content, RS content could also be affected by amylose–lipid complexes and the degree of crystallinity [23]. The high content of resistant starch and amylose in purple sweet potatoes indicated a potential to produce resistant starch. After enzymatic hydrolysis, the WBC of PSPRS significantly decreased from 283.74% to 186.78%, respectively (*p* < 0.05). These may be due to the decrease of hydrophilic groups and the increase of hydrophobic groups after treatment with amylase.

### 3.2. Color Parameters

Color parameters (*L*, *a*, and *b*) are represented in Table 2. The whiteness of PSPS and PSPRS was greater than 90. PSPS and PSPRS both showed a slight greenish tint and yellowness in color, which might be due to the co-pigmented substance between anthocyanin and protein [13].

### 3.3. Morphological Characteristics

**Figure 1:**
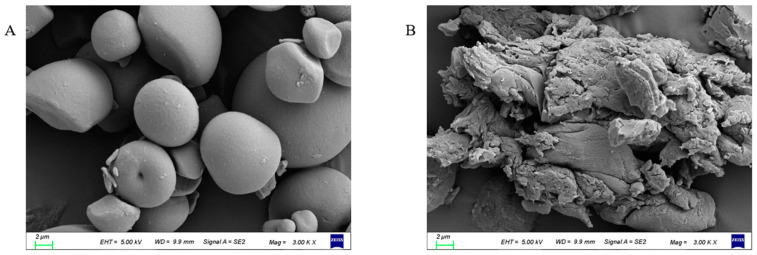
SEM images of PSPS (**A**) and PSPRS (**B**) (magnification, 3000×).

The surface characteristics and shapes of PSPS and PSPRS were observed by SEM (Figure 1). The PSPS granules were smooth-surfaced, round, irregular, and elliptical, which is in agreement with the report of Yong et al. [37]. Compared with PSPS, the outward structure of the PSPRS granules was oval in shape and irregular multi-layer stacking fragments with a rough surface and larger amounts of cracks and cavities. Liu et al. [38] also found that enzymatically hydrolyzed starch showed signs of cracks and a rough surface. The structure of the PSPRS granule might lead to microbial community responses during colonic fermentation in vivo.

### 3.4. Structural Characterization

**Figure 2:**
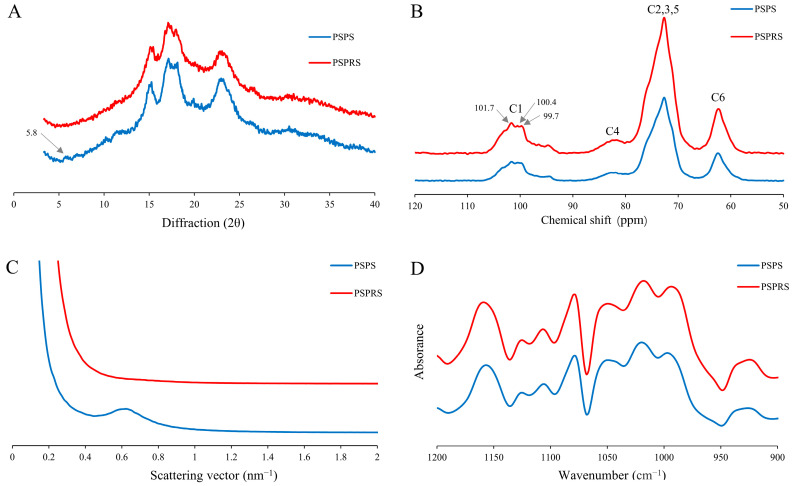
X-ray diffraction pattern (**A**); solid-state ^13^C CP/MAS NMR spectra (**B**); SAXS pattern (**C**); and deconvoluted FTIR spectra (**D**) of PSPS and PSPRS.

The XRD patterns of PSPS and PSPRS are displayed in Figure 2A. According to XRD patterns, PSPS had the characteristics of C_A_-type crystalline with diffraction peaks at 15° and 23° 2θ, unresolved doublet peaks at approximately 17° and 18° 2θ, and a small peak at closely 5.8° 2θ, based on a previous study by Guo et al. [21]. The disappearance of the peak at 5.8° 2θ and the decrease in peak intensity at 15°, 23°, 17°, and 18° 2θ of PSPRS indicate that the crystalline structure changed from C_A_-type to A-type during enzymatic hydrolysis. The results revealed that the B-type allomorph in C-type starch was degraded faster than the A-type allomorph in the process during enzymatic hydrolysis. Moreover, the relative crystallinity of PSPRS (30.53%) was lower than that of PSPS (Table 2), which may be due to the higher amylose content in PSPRS. Costa et al. [23] also found that relative crystallinity was inversely associated with amylose content, which is consistent with our results.

The characterization of crystalline polymorphic starch was analyzed by solid-state ^13^C CP/MAS NMR [37]. According to the literature, signals at 94–105 ppm were related to C1, signals at 80–84 ppm appeared from the amorphous domains for C4, overlapping signals at 68–78 ppm were assigned to C2, C3, and C5, and signals at 58–65 ppm were attributed to C6 [39]. A-type starch presents triplet peaks at approximately 102, 101, and 100 ppm; B-type starch exhibits doublet peaks at about 101 and 100 ppm; while C-type starch shows a mixed pattern that depends on the relative proportions of A- or B-type polymorphs [40]. Figure 2B shows the 13C CP/MPS NMR spectra of PSPS and PSPRS. The PSPS has triplet peaks at 101.6, 100.4, and 99.7 ppm, suggesting the character of C_A_-type starch. However, the C1 resonance of PSPRS gradually became a clear triplet at the same peaks, corresponding to the characteristics of A-type crystallinity. This result was in agreement with that of XRD, further confirming PSPRS had A-type crystallinity.

As shown in Figure 2C and Table 2, the main scattering peak (S_max_) for PSPS is at 0.626 nm^−1^. However, the typical peak at 0.6–0.7 nm^−1^ cannot be observed in PSPRS, which was replaced by a nearly straight line. This demonstrated that the lamellar structure in the PSPRS granule was totally destroyed during enzymatic hydrolysis. In addition, the scatting peak intensity (I_max_) was proportional to the size of non-homogeneous areas within the starch samples [41]. The I_max_ in PSPS was 187.138, whereas it disappeared in PSPRS, possibly due to the hydrolysis of semi-crystalline growth rings. The SAXS patterns were consistent with the changes in morphology (Figure 1B).

FTIR spectra in the range of 1200–900 cm^−1^ of PSPS and PSPRS were shown in Figure 2D. The bands at 1047 cm^−1^ and 1022 cm^−1^ are sensitive to the crystalline regions and amorphous regions of starch, respectively. The ratio of absorbance (1047/1022 cm^−1^) is frequently applied to quantify the ordered degree of starch. Jiang et al. [42] found that a higher ordered degree in the starch granules corresponds to a higher proportion of crystallinity. The ratio of the absorbance 1047/1022 cm^−1^ of PSPS and PSPRS was 0.723 and 0.689, respectively (Table 2). The crystalline region in PSPS was higher than in PSPRS, and this finding was in agreement with the results of crystallinity in the XRD patterns.

### 3.5. Effects of PSPRS on Immune Organ Indices

Body weight loss is a common symptom of DSS-induced colitis [43]. As shown in Table 3, the initial body weight of mice was not statistically different between the four groups. The final body weight in the DSS group was significantly lower in comparison to the NC group. After the intervention with PSPRS, the body weights were recovered, particularly in the high-dose PSPRS group. A significant decrease in the thymus index was observed in the DSS group compared to the NC group (*p* < 0.05), whereas the spleen index denoted an opposite trend, indicating the colitis model was successfully established. There was no significant difference in the thymic index between the DSS group and the DSS+LPSPRS group (*p* > 0.05). However, the thymic index of the DSS+HPSPRS group was significantly improved in comparison with the DSS group (*p* < 0.05). Significantly, both 100 mg/kg and 300 mg/kg of PSPRS showed a protected effect on spleen enlargement in colitis mice, and 300 mg/kg of PSPRS was superior to 100 mg/kg of PSPRS. The thymus is a major organ that produces T cells, while the spleen could remove foreign body bacteria in the blood and produce immune substances [44]. When the mice suffer from exogenous instigation, including DSS, the thymus is prone to pathological atrophy. Additionally, the DSS stimulus can activate cellular immune responses, leading to enlargement of the spleen. The thymus and spleen indexes reflect innate immunity function [45]. Indeed, these changes in spleen and thymus indices reflect disorders in immunity. The consumption of PSPRS could alleviate excessive inflammatory immune responses and protect the immune organs. The above results propose that the intervention with PSPRS can protect against the loss of weight, thymus atrophy, and spleen hypertrophy induced by DSS.

### 3.6. Histological Characterization

**Figure 3:**
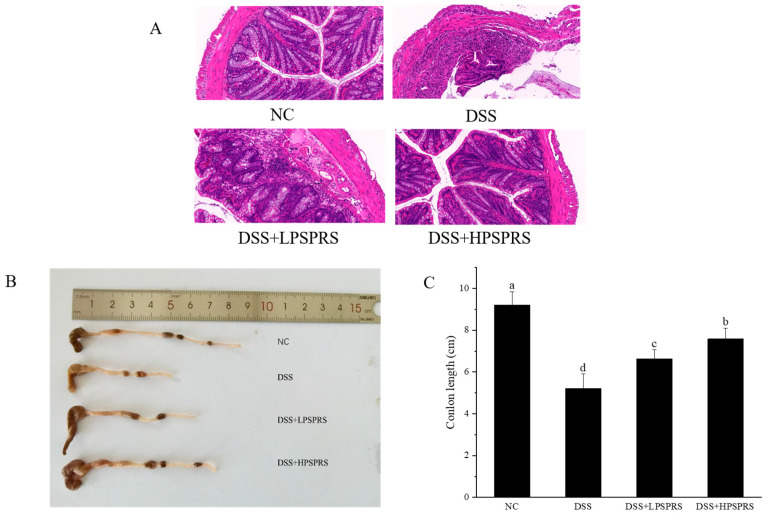
Effects of PSPRS on the colon. Histological analysis (**A**); photos of colon appearance (**B**); and values of colon length in four groups were expressed as the mean ± SD (n = 6). Data in the same column with different letters were significantly different at the level of *p* < 0.05 (**C**).

The H&E staining results are displayed in Figure 3A. The mice in the NC group had distinct crypt structures, rich goblet cells, and neatly arranged glands. However, the DSS caused an absence of goblet cells, infiltration of inflammatory cells, and reduction of crypts. The intervention with PSPRS relieved these symptoms. In particular, intragastric administration of 300 mg/kg of PSPRS restored the epithelial barrier structure and reduced inflammatory cell infiltration. The length of the colon is inversely proportional to the severity of colonic inflammation [46]. Histological analysis more intuitively illustrated that PSPRS has a protective effect on inflammatory infiltration of colon tissue. Inflammation and tissue integrity are indirectly measured by colon length [47]. As indicated in Figure 3B,C, the average colon length in the DSS group was notably shorter than that in the NC group by 40.88% due to serious colitis. The 100 mg/kg and 300 mg/kg doses of PSPRS ameliorated DSS-induced colon shortening. These results revealed that PSPRS had a protective effect on DSS-induced colitis mice.

### 3.7. Effects of PSPRS on Inflammatory Cytokines in DSS-Induced Colitis Mice

Inflammatory cytokine levels indicate the extent of inflammation in the intestinal lining [48]. To further evaluate the regulatory effects of PSPRS on DSS-induced colitis mice, the levels of several cytokines in mouse colon tissue are presented in Table 4. Pro-inflammatory cytokines, including IL-6, IL-1β, and TNF-α, in mouse colon tissue were all significantly increased in the DSS group compared to the NC group (*p* < 0.05). However, IL-1β, IL-6, and TNF-α levels in the colon tissues were significantly inhibited after the intervention with PSPRS (*p* < 0.05). Among them, the levels of IL-6, IL-1β, and TNF-α in the DSS+HPSPRS group were observably lower than those in the DSS+LPSPS group (*p* < 0.05). Compared with the NC group, the levels of the anti-inflammatory cytokine IL-10 were significantly reduced in DSS-induced colitis mice (*p* < 0.05). Whereas, the level of IL-10 was increased by administration of PSPRS (*p* < 0.05), and 300 mg/kg of PSPRS showed a better effect than 100 mg/kg of PSPRS. The DSS+LPSPRS and DSS+HPSPRS groups showed an increasing trend in the level of IgA compared with the DSS group. Our results indicated PSPRS had a regulatory role in inflammatory cytokines in a dose-dependent manner.

Inflammation is an important body-defense mechanism that can effectively restore tissue damage. The inflammatory process is mainly regulated and mediated by several pro-inflammatory and anti-inflammatory cytokines [49]. TNF-α is a key mediator for UC development and is mainly produced by macrophages and monocytes [50]. Excessive TNF-α can enhance intestinal permeability, induce cell apoptosis, damage the intestinal barrier, and activate the NF-κB pathway, along with increasing the secretion of IL-1β and IL-6 [51,52]. Moreover, IL-6 can also promote the development of colitis, not only controlling the proliferation and survival of T cells but also stimulating IL-1 and IL-17 secretion [53]. Interestingly, IL-6 has anti-inflammatory activities like those of IL-10 through a different mechanism. IL-10, as one of the most important anti-inflammatory cytokines, can inhibit the secretion of TNF-α, IL-6, and IL-1β and reduce antigen presentation [54]. Immunoglobulin A (IgA) helps to maintain intestinal homeostasis by decreasing mucosal surface invasion by pathogens and strengthening intestinal barrier function [55]. Therefore, the present study suggested that administration of PSPRS protected mice against DSS-induced colitis by decreasing the levels of TNF-α, IL-6, and IL-1β and elevating the production of IL-10 and IgA. Previous studies have reported that green banana-resistant starch also regulates pro- and anti-inflammatory cytokines to attenuate DSS-induced colitis in mice [56].

### 3.8. Effects of PSPRS on the Production of SCFAs in DSS-Induced Colitis Mice

Colonic microbiota can use dietary fiber to produce SCFAs that serve as potent anti-inflammatory agents. RS can be easily fermented into SCFAs by the gut microbiota. It has been reported that SCFAs, including acetate, butyrate, and propionate, have effects on the prevention and treatment of patients with IBD [12]. Therefore, we further evaluated the modulatory effect of PSPRS on gut microbiota and SCFAs in DSS-induced colitis mice. Based on the results of PSPRS action on organ index, colon tissue pathology, and inflammatory factors, we found that the anti-inflammatory effect of 300 mg/kg of PSPRS was significantly higher than that of 100 mg/kg of PSPRS. Therefore, the 300 mg/kg PSPRS group was applied in the subsequent research on gut microbiota and short-chain fatty acids.

As shown in Table 5, acetate, propionate, and butyrate levels in the DSS group were obviously lower than those in the NC group (*p* < 0.05). 300 mg/kg of PSPRS administration can significantly improve the production of acetate, propionate, and butyrate (*p* < 0.05). SCFAs can act on FOXP3^+^ T cells to produce cytokines such as TGF-β and IL-10 and inactivate the NF-kB signaling pathway [57]. Butyrate, as the main energy source for colonic mucosal enterocytes, has been proven to suppress inflammation in UC development [58]. Qian et al. [59] reported that the fermentation of SCFAs by the gut microbiota is related to the type of RS, and different types of RS have different structural characteristics, ultimately leading to SCFA production. Our results suggest that the anti-inflammatory effect of PSPRS might be enhanced by SCFA contents, especially the levels of butyrate.

### 3.9. Effect of PSPRS on the Gut Microbiota in DSS-Induced Colitis Mice

#### 3.9.1. Operational Taxonomic Unit (OTU) Analysis

**Figure 4:**
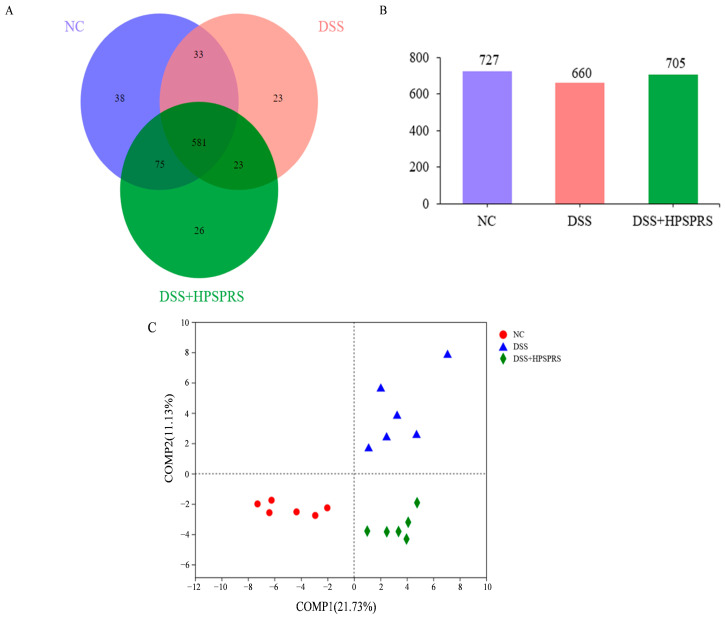
Venn diagram of OTUs (**A**); comparison of the total OTUs (**B**); partial least squares discriminant analysis (PLSDA) of the gut microbiota in each group (**C**).

OTUs in the feces of mice can reflect the gut microbiota diversity [60]. According to the Venn diagram in Figure 4A, 75 OTUs were shared by the NC and 300 mg/kg PSPRS groups, indicating that the gut microbiota composition of the DSS-induced colitis mice after PSPRS administration was more similar to that of normal control mice as compared to the DSS group. Additionally, the 581 OTUs are shared by three groups of mice. The total number of OTUs in three groups is displayed in Figure 4B. OTUs in the NC group, DSS group, and DSS+300 mg/kg of PSPRS group were 727, 660 and 705, respectively, indicating that the consumption of PSPRS groups could recover the reduced OTUs induced by DSS.

#### 3.9.2. PLS-DA Analysis

To determine whether the overall structural change of gut microbiota in three groups was significant, we performed PLS-DA analysis. As shown in Figure 4C, the NC group, the DSS group, and the DSS+300 mg/kg PSPRS group were located at the lower left hand, upper right, and lower right sides of the figure, respectively. The structure of the gut microbes was significantly altered by DSS, but consumption of 300 mg/kg of PSPRS markedly adjusted the gut microbes in DSS-induced colitis mice.

#### 3.9.3. Alpha Diversity Analysis

Alpha diversity in the gut has emerged as a potential indicator of overall health [61]. Shannon, Simpson, ACE, and coverage indices were used to evaluate the diversity and richness of the microflora [62]. As shown in Table 6, the DSS group showed an obvious reduction of the Sobs, Shannon, and ACE indices and an obvious augment of the Simpson index when compared to the NC group (*p* < 0.05). Alpha diversity was reversed by PSPRS treatment compared with DSS treatment. Notably, the Sobs and Shannon indices were significantly increased while the Simpson index decreased when compared to the DSS group (*p* < 0.05). Additionally, the nearly average value of 0.999 of the coverage index was large enough to reflect most of the microbial diversity information in the sample. Tong et al. [63] reported that alpha diversity was found to increase with the improvement of symptoms in DSS-induced colitis. The above results reaffirm that PSPRS treatment effectively improved colitis.

#### 3.9.4. Composition and Abundance of Gut Microflora at the Phylum Level

**Figure 5:**
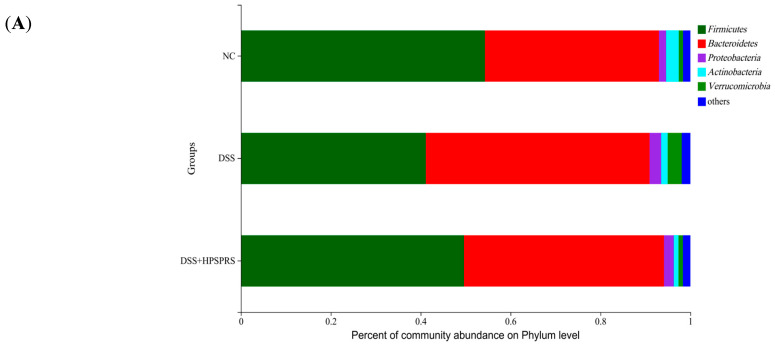

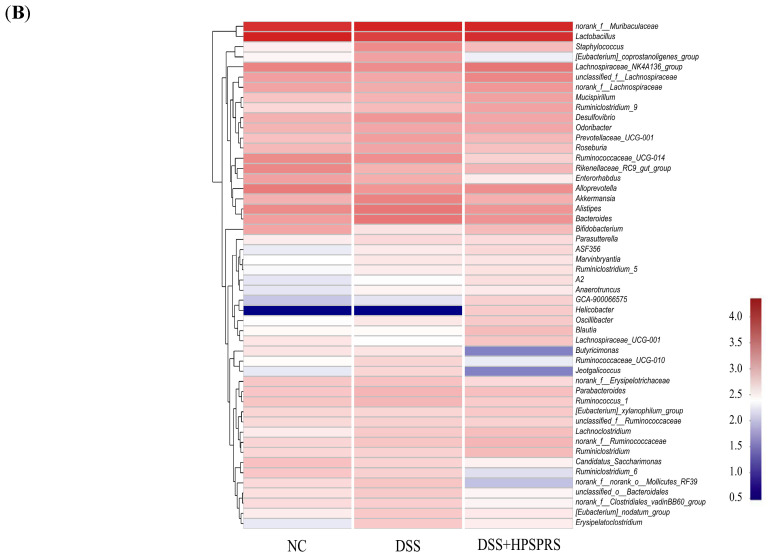
The relative abundances of gut microbiota at the level of phylum (**A**) and genus (**B**) in three groups. Data are expressed as mean ± SD (n = 6).

To further investigate the changes in microbial species, phylum levels of gut microbiota were analyzed. As shown in Figure 5A and Table 7, *Firmicutes*, *Bacteroidetes*, *Proteobacteria*, *Actinobacteria*, and *Verrucomicrobia* were the five relative predominant abundances of microbes at the phylum level. The gut microbiota in patients with IBD was primarily characterized by a reduced ratio of *Firmicutes* and *Bacteroidetes* [12]. On the contrary, the increased ratio is usually thought to contribute to alleviating IBD [64]. We found that 300 mg/kg of PSPRS significantly mitigated the decline in the ratio of *Firmicutes* and *Bacteroidetes* caused by DSS (*p* < 0.05). The imbalanced gut microbiota and potential risk of colitis are often caused by the unusual expansion of *Proteobacteria*. PSPRS significantly inhibited the development of *Proteobacteria*. Recent research found the expansion of *Verrucomicrobia* was detected when treated with DSS, which was consistent with our findings [65]. However, PSPRS administration reduced the proportion of *Verrucomicrobia* (*p* < 0.05). The above results suggested that PSPRS possessed a mitigating effect on DSS-induced colitis.

#### 3.9.5. Analysis of Gut Microflora at the Genus Level

The composition of the gut microbiota at the genus level was shown in Figure 5B. It can be seen that the relative abundance in each group mainly includes *norank_f_Muribaculaceae*, *Lactobacillus*, *Lachnospiraceae_Nk4A136_group*, *Alistipes*, *Bacteroides*, *Alloprevotella*, *unclassified_f_Lachnospiraceae*, *Staphylococcus*, and *Akkermansia*. As indicated in Table 8, the relative abundance of *Bacteroides*, *Staphylococcus*, *Akkermansia*, *Desulfovibrio*, *Jeotgalicoccus*, and *Erysipelatoclostridium* was significantly enhanced (*p* < 0.05), while the relative abundances of *Lactobacillus*, *Lachnospiraceae_NK4A136_group*, *Alloprevotell*, and *Bifidobacterium* were notably reduced in DSS-induced colitis mice when compared to the NC group (*p* < 0.05). Whereas, the disorder of gut microbiota induced by DSS was restored by PSPRS treatment, which significantly inhibited the decrease of *Lactobacillus* and *Lachnospiraceae_NK4A136_group* as well as the increase of *Bacteroides*, *Staphylococcus*, *Akkermansia*, *Jeotgalicoccus*, and *Erysipelatoclostridium* (*p* < 0.05). Moreover, the relative level of *Alistipes* in the DSS+HPSPRS group was notably declining when compared to the DSS group (*p* < 0.05). The abundance of *Alloprevotella* was positively related to the production of SCFAs but negatively related to TLR4 signaling and LPS levels [66]. *Birfdobacterium* can produce butyrate, which provides an anti-inflammatory effect [67]. However, all of *Bacteroides*, *Desulfovibrio*, *Staphylococcus*, and *Jeotgalicoccus* are often believed to be positively correlated with inflammation, which can aggravate colitis [68,69,70]. Some studies have proposed that *Alistipes* has been known as a colitogenic strain, which is enhanced in IBD patients [71]. Zhang et al. [72] found that *Akkermansia* is a mucin-degrading commensal that could decrease the content of SCFAs and increase the secretion of pro-inflammatory factors. Previous studies suggested the abundance of *Erysipelatoclostridium* was enhanced in DSS-induced colitis mice [73]. PSPRS might alleviate intestinal inflammation by increasing the relative abundance of *Lactobacillus*, *Alloprevotella*, *Lachnospiraceae_NK4A136_group*, and *Birfdobacterium* and decreasing the relative abundance of *Bacteroides*, *Staphylococcus*, *Akkermansia*, *Jeotgalicoccus*, *Erysipelatoclostridium*, and *Alistipes* [74].

**Figure 6:**
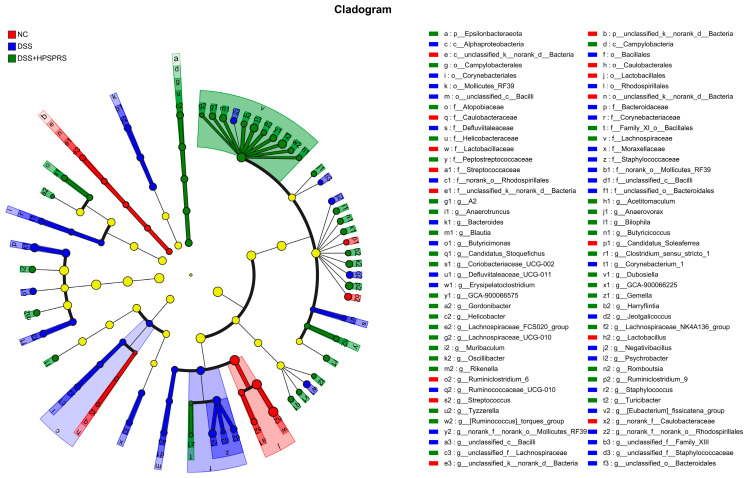
LEfSe analysis of HPSPRS intervention on the gut microbiota.

Linear discriminant analysis effect size (LEfSe) analysis was further used to analyze the different abundant taxa among the three groups. As shown in Figure 6, the cladogram was demonstrated at the taxonomic level, from phylum to genus. There were 84 differential taxa in the three groups. Among them, 15, 32, and 37 taxa were enriched in the NC group, DSS group, and DSS+HPSPRS group, respectively, indicating each group all harbored different dominant taxa. In addition, the bacterial taxa at the genus level of *Staphylocc*ous, *Jeotgalicoccu*, *Bacteroides*, and *Erysipelatoclostridium* were expanded in the DSS group. Nevertheless, this trend was reversed by the PSPRS administration. Furthermore, the relative abundances of *Lachnospiraceae_NK4A136_group* and *unclassified-f-lachnospiraceae* increased after PSPRS intervention. The results indicated the gut microbiota composition could be well modulated in DSS-induced colitis mice following administration of PSPRS.

#### 3.9.6. Correlations between the Gut Microbiota and UC Parameters

**Figure 7:**
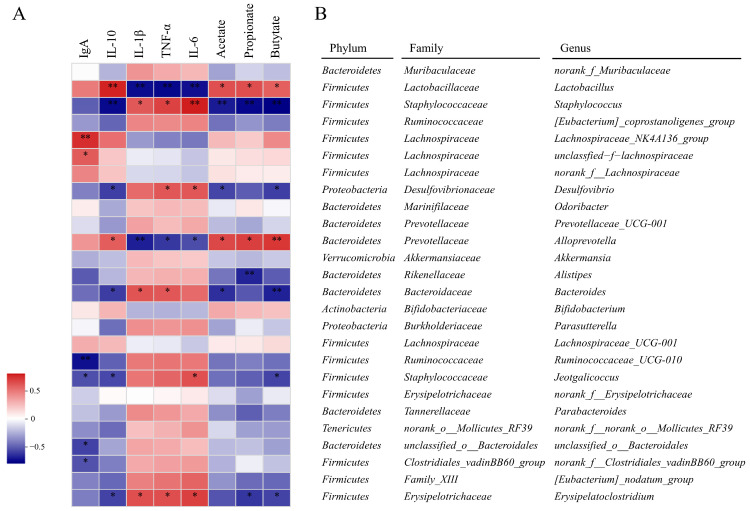
Heatmap of correlations between the gut microbiota at the genus level and UC parameters (**A**). Red squares indicate positive correlation, and blue squares indicate negative correlation. * and ** indicate significant differences (*p* < 0.05) and extremely significant differences (*p* < 0.01), respectively. Information annotation of gut microbiota at the genus level (**B**).

A heatmap of the Spearman correlation between 26 genera reversed by PSPRS intervention and UC-related indexes (inflammatory cytokines, IgA, and SCFAs) was generated to evaluate the pathogenesis of UC. As shown in Figure 7, 13 kinds of genus were remarkably correlated with at least one physical index. To be specific, *Staphylococcus*, *Desulfovibrio*, *Bacteroides*, and *Jeotgalicoccus Erysipelatoclostridium* were positively correlated with parameters promoting colitis and negatively correlated with parameters preventing colitis. Similarly, *Ruminococcaceae_UCG-010*, *unclassified_o_Bacteroidales*, *unclassified_o_Bacteroidales*, and *norank_f_Clostridiales_vadinBB60_group* were positively correlated with parameters promoting colitis. Furthermore, negative correlations with parameters that prevent colitis were found with *Alistipes*. Gut bacteria, whose relative abundances were increased after PSPRS administration, were found to be positively correlated with parameters preventing colitis and negatively correlated with parameters promoting colitis. Specifically, *Lactobacillus* and *Alloprevotella* were highly negatively correlated parameters preventing colitis. In addition, it was observed that *Lachnospiraceae_NK4A136_group* and *unclassified-f-lachnospiraceae* were highly positively correlated with parameters preventing colitis. The above results suggested that the protective effects of PSPRS on DSS-induced colitis mice may work by suppressing pro-inflammatory cytokines and increasing SCFA production and anti-inflammatory cytokines.

### 3.10. Analysis of 16S Function Prediction

**Figure 8:**
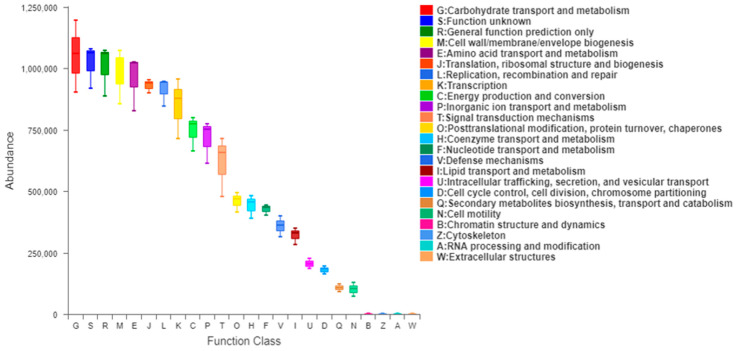
Effect of HPSPRS on COG function classification of the gut microbiota predicted by PICRUSt.

As shown in Figure 8, the 16S amplicon sequencing results were predicted by PICRUSt, and we obtained the clusters of orthologous groups (COG) function classification of the gut microbiota among each group. The results showed that the functions involved carbohydrate transport and metabolism, general function prediction only, amino acid transport and metabolism, cell wall/membrane/envelop biogenesis, transcription, energy production and conversion, defense mechanisms, and so on. Moreover, the PSPRS especially caused changes in carbohydrate transport and metabolism (predominantly lipid metabolism, amino acid metabolism, and human disease pathways). In summary, the results indicated that the PSPRS contributed to the functional variation of the gut microbiota and/or its metabolites.

Previously, starches from potato, pea, and Chinese yam have been shown to alleviate DSS-induced colitis symptoms and also show significant prebiotic characteristics [75]. Starch from *Pueraria lobata* demonstrated gut microbiota-dependent protection against colitis [76]. Polysaccharide from *Arctium lappa* and yellow sweet potato is effective in protecting mice from DSS-induced colitis [77,78]. The results of the present study demonstrated that PSPRS alleviated DSS-induced colitis via attenuating inflammation and maintaining gut homeostasis. The relationship between microbiota and PSPRS structure remains unclear. Further investigations, like the fecal metabolome, are warranted to elucidate the intricate relationship between colonic microorganisms and PSPRS structure. In addition, a single dose of PSPRS was used on gut microbiota and SCFAs in DSS-induced colitis mice; more doses should be chosen to comprehensively clarify the anti-inflammatory effect of PSPRS in future research. In addition, the relationship between the structural characteristics of PSPRS and its physiological functions needs to be further investigated. DSS-induced mouse models and human colitis may cause disparities in physiology; future clinical trials and various cohorts of mice are needed to confirm the therapeutic efficacy of PSPRS.

## 4. Conclusions

Taken together, our results showed that consumption of PSPRS could protect mice from DSS-induced colitis. Various colitis symptoms in mice, including body weight, thymus index, spleen index, IL-6, TNF-α, IL-1β, IL-10, and IgA, were restored with 100 mg/kg PSPRS and 300 mg/kg PSPRS administration. PSPRS recovered the disordered gut microbiota and improved SCFA generation. Those changes in microbiota and SCFAs could enhance the intestinal barrier by restricting goblet cell loss and intestinal epithelial cell apoptosis. All these changes that happened in mice could help protect DSS-induced colitis mice. Therefore, PSPRS might be considered an effective dietary strategy for treating or preventing UC. In the future, human volunteer experiments and various cohorts of mouse models can be carried out to reveal the exact anti-inflammatory mechanisms of PSPRS.

## Figures and Tables

**Table 1:** Moisture, ash, protein, starch, amylose, resistant starch water-binding capacity (WBC), and paste clarity content of PSPS and PSPRS. Except for moisture content, other traits were expressed in dry basis.

Sample ^1^	Moisture (%)	Ash (%)	Protein (%)	Starch (%)	Amylose (%)	Resistant Starch (%)	WBC (%)
PSPS	9.38 ± 0.35 ^a^	1.07 ± 0.43 ^a^	0.85 ± 0.08 ^a^	92.30 ± 0.51 ^a^	24.42 ± 0.61 ^b^	28.12 ± 1.25 ^b^	283.74 ± 4.73 ^a^
PSPRS	7.79 ± 0.13 ^b^	1.38 ± 0.29 ^a^	0.06 ± 0.04 ^b^	89.83 ± 0.58 ^b^	38.69 ± 0.53 ^a^	71.64 ± 2.67 ^a^	222.48 ± 3.27 ^b^

^1^ Values are expressed as the mean ± SD (n = 3). Data in the same column with different superscripts were significantly different at the level of *p* < 0.05.

**Table 2:** The color parameters, relative crystallinity, and SAXS parameters of PSPS and PSPRS.

Sample	Color Parameters ^1^			Relative Crystallinity	SAXS Parameter ^2^			IR Ratio of 1047 cm^−1^/1022 cm^−1^
	*L*	*a*	*b*		S_max_ (nm^−1^)	d (nm)	I_max_	
PSPS	95.28 ± 0.42 ^a^	−1.24 ± 0.14 ^a^	3.70 ± 0.57 ^a^	34.78	0.626	10.031	187.138	0.723
PSPRS	91.78 ± 0.98 ^b^	−0.69 ± 0.05 ^b^	4.52 ± 0.73 ^a^	30.51	-- ^3^	--	--	0.689

^1^ Values are expressed as the mean ± SD (n = 3). Data in the same column with different superscripts were significantly different at the level of *p* < 0.05. ^2^ SAXS parameters of S_max_, main scattering peak position; d Bragg spacing (2π/S_max_); I_max_, the scatting peak intensity. ^3^ Data were not detected.

**Table 3:** Effects of PSPRS on the body weight, thymus index, and spleen index.

Sample Groups ^1^	Initial Body Weight (g)	Final Body Weight (g)	Thymus Index (mg/g)	Spleen Index (mg/g)
NC	30.07 ± 0.71 ^a^	36.22 ± 1.44 ^a^	1.57 ± 0.10 ^a^	3.08 ± 0.19 ^c^
DSS	30.47 ± 0.62 ^a^	31.36 ± 1.09 ^c^	1.10 ± 0.10 ^c^	3.84 ± 0.16 ^a^
DSS+LPSPRS	30.13 ± 0.70 ^a^	32.64 ± 0.53 ^bc^	1.22 ± 0.06 ^c^	3.55 ± 0.09 ^b^
DSS+HPSPRS	30.58 ± 0.93 ^a^	34.08 ± 0.78 ^b^	1.34 ± 0.04 ^b^	3.25 ± 0.16 ^c^

^1^ Values are expressed as the mean ± SD (n = 6). Data in the same column with different superscripts were significantly different at the level of *p* < 0.05. NC: normal control; DSS: DSS model control; DSS+LPSPRS: 100 mg/kg PSPRS+DSS; and DSS+HPSPRS: 300 mg/kg PSPRS+DSS.

**Table 4:** The levels of inflammatory cytokines and sIgA in the colons of mice.

Sample Groups ^1^	IL-6 (pg/mL)	TNF-α (pg/mL)	IL-1β (pg/mL)	IL-10 (pg/mL)	IgA (µg/mL)
NC	54.17 ± 4.71 ^d^	501.64 ± 40.77 ^d^	67.53 ± 7.42 ^d^	620.93 ± 33.39 ^a^	3.15 ± 0.18 ^ab^
DSS	97.86 ± 8.03 ^a^	856.81 ± 50.64 ^a^	114.13 ± 4.53 ^a^	256.47 ± 13.85 ^d^	2.63 ± 0.12 ^c^
DSS+LPSPRS	75.33 ± 4.71 ^b^	704.88 ± 27.61 ^b^	101.94 ± 5.78 ^b^	361.37 ± 28.13 ^c^	2.85 ± 0.18 ^bc^
DSS+ HPSPRS	64.53 ± 4.90 ^c^	629.35 ± 35.79 ^c^	87.95 ± 8.99 ^c^	540.26 ± 26.82 ^b^	3.33 ± 0.31 ^a^

^1^ Values are expressed as the mean ± SD (n = 6). Data in the same column with different superscripts were significantly different at the level of *p* < 0.05. NC: normal control; DSS: DSS model control; DSS+LPSPRS: 100 mg/kg PSPRS+DSS; and DSS+HPSPRS: 300 mg/kg HPSPRS+DSS.

**Table 5:** Effects of HPSPRS on the levels of acetate, propionate, and butyrate.

Sample Groups ^1^	Acetate	Propionate	Butyrate
NC	6.35 ± 0.78 ^a^	0.86 ± 0.18 ^a^	1.02 ± 0.36 ^a^
DSS	2.32 ± 0.43 ^c^	0.27 ± 0.07 ^c^	0.27 ± 0.12 ^b^
DSS+ HPSPRS	3.96 ± 0.39 ^b^	0.57 ± 0.11 ^b^	0.74 ± 0.13 ^a^

^1^ Values are expressed as the mean ± SD (n = 6). Data in the same column with different superscripts were significantly different at the level of *p* < 0.05. NC: normal control; DSS: DSS model control; and DSS+HPSPRS: 300 mg/kg PSPRS+DSS.

**Table 6:** Alpha diversity index of mice in each group.

Sample Groups ^1^	Sobs	Shannon	Simpson	ACE	Coverage
NC	110.00 ± 3.79 ^a^	2.62 ± 0.27 ^a^	0.17 ± 0.038 ^b^	113.49 ± 5.01 ^a^	0.9995 ± 0.0002 ^a^
DSS	95.30 ± 3.35 ^c^	1.93 ± 0.25 ^b^	0.31 ± 0.061 ^a^	104.47 ± 4.39 ^b^	0.9994 ± 0.0005 ^a^
DSS+HPSPRS	104.30 ± 3.59 ^b^	2.52 ± 0.31 ^a^	0.19 ± 0.052 ^b^	109.37 ± 3.67 ^ab^	0.9995 ± 0.0001 ^a^

^1^ Values are expressed as the mean ± SD (n = 6). Data in the same column with different superscripts were significantly different at the level of *p* < 0.05.

**Table 7:** Relative abundance of predominant taxa at the phylum level.

Sample Groups ^1^	*Firmicutes* (%)	*Bacteroidetes* (%)	*Proteobacteria* (%)	*Actinobacteria* (%)	*Verrucomicrobia* (%)	The Ratio of *Firmicutes* to *Bacteroidetes*
NC	54.29 ± 5.78 ^a^	38.69 ± 5.40 ^b^	1.62 ± 0.39 ^b^	2.83 ± 1.30 ^a^	0.92 ± 0.54 ^b^	1.42 ± 0.19 ^a^
DSS	41.14 ± 4.25 ^b^	49.78 ± 6.74 ^a^	2.59 ± 0.32 ^a^	1.45 ± 0.85 ^ab^	3.12 ± 1.69 ^a^	0.85 ± 0.16 ^c^
DSS+HPSPRS	49.63 ± 3.69 ^a^	44.49 ± 3.06 ^ab^	2.10 ± 0.37 ^ab^	1.04 ± 0.54 ^b^	0.97 ± 0.77 ^b^	1.12 ± 0.12 ^b^

^1^ Values are expressed as the mean ± SD (n = 6). Data in the same column with different superscripts were significantly different at the level of *p* < 0.05.

**Table 8:** Relative abundance of taxa at the genus level.

Gut Microbiota ^1^	Relative Abundance (%)			Dynamic Change in Relative Abundance ^2^	
	NC	DSS	DSS+HPSPRS	DSS vs. NC	DSS+HPSPRS vs. DSS
*norank_f_Muribaculaceae*	25.86 ± 6.26 ^a^	34.66 ± 9.17 ^a^	33.67 ± 7.55 ^a^	↑	↓
*Lactobacillus*	36.87 ± 6.63 ^a^	17.64 ± 5.36 ^c^	26.89 ± 3.42 ^b^	↓ *	↑ *
*Staphylococcus*	0.17 ± 0.10 ^b^	2.40 ± 0.51 ^a^	0.69 ± 0.22 ^b^	↑ *	↓ *
*[Eubacterium]_coprostanoligenes_group*	0.14 ± 0.09 ^ab^	1.39 ± 1.27 ^a^	0.07 ± 0.04 ^b^	↑	↓ *
*Lachnospiraceae_NK4A136_group*	3.09 ± 0.26 ^b^	2.41 ± 0.38 ^c^	4.62 ± 0.84 ^a^	↓ *	↑ *
*unclassfied_f_lachnospiraceae*	1.45 ± 0.47 ^b^	1.17 ± 0.56 ^b^	2.84 ± 0.77 ^a^	↓	↑ *
*norank_f_Lachnospiraceae*	1.31 ± 0.21 ^ab^	1.06 ± 0.54 ^b^	1.83 ± 0.46 ^a^	↓	↑ *
*Desulfovibrio*	0.93 ± 0.29 ^b^	1.88 ± 0.51 ^a^	1.23 ± 0.25 ^ab^	↑ *	↓
*Odoribacter*	0.84 ± 0.16 ^a^	1.23 ± 0.59 ^a^	1.22 ± 0.46 ^a^	↑	↓
*Prevotellaceae_UCG-001*	0.60 ± 0.40 ^a^	1.52 ± 0.96 ^a^	0.77 ± 0.45 ^a^	↑	↓
*Alloprevotella*	3.90 ± 1.12 ^a^	1.76 ± 0.67 ^b^	2.31 ± 0.55 ^ab^	↓ *	↑
*Akkermansia*	0.92 ± 0.55 ^b^	3.12 ± 1.69 ^a^	0.97 ± 0.77 ^b^	↑ *	↓ *
*Alistipes*	2.57 ± 0.97 ^ab^	4.33 ± 1.73 ^a^	1.90 ± 0.78 ^b^	↑	↓ *
*Bacteroides*	1.50 ± 0.71 ^b^	4.36 ± 1.32 ^a^	2.10 ± 0.45 ^b^	↑ *	↓ *
*Bifidobacterium*	1.27 ± 0.51 ^a^	0.24 ± 0.15 ^b^	0.69 ± 0.34 ^ab^	↓ *	↑
*Parasutterella*	0.18 ± 0.05 ^a^	0.31 ± 0.16 ^a^	0.29 ± 0.09 ^a^	↑	↓
*Lachnospiraceae_UCG-001*	0.22 ± 0.15 ^ab^	0.10 ± 0.04 ^b^	0.55 ± 0.34 ^a^	↓	↑ *
*Ruminococcaceae_UCG-010*	0.12 ± 0.06 ^b^	0.36 ± 0.17 ^a^	0.06 ± 0.02 ^b^	↑ *	↓ *
*Jeotgalicoccus*	0.06 ± 0.05 ^b^	0.34 ± 0.26 ^a^	0.01 ± 0.01 ^b^	↑ *	↓ *
*norank_f_Erysipelotrichaceae*	0.50 ± 0.32 ^a^	0.57 ± 0.21 ^a^	0.32 ± 0.19 ^a^	↑	↓
*Parabacteroides*	0.51 ± 0.25 ^a^	0.85 ± 0.27 ^a^	0.62 ± 0.24 ^a^	↑	↓
*norank_f_norank_o_Mollicutes_RF39*	0.31 ± 0.11 ^b^	0.54 ± 0.10 ^a^	0.02 ± 0.01 ^c^	↑ *	↓ *
*unclassified_o_Bacteroidales*	0.26 ± 0.11 ^b^	0.46 ± 0.07 ^a^	0.14 ± 0.12 ^b^	↑ *	↓ *
*norank_f_Clostridiales_vadinBB60_group*	0.29 ± 0.19 ^a^	0.42 ± 0.23 ^a^	0.14 ± 0.07 ^a^	↑	↓
*[Eubacterium]_nodatum_group*	0.18 ± 0.13 ^a^	0.47 ± 0.28 ^a^	0.20 ± 0.09 ^a^	↑	↓
*Erysipelatoclostridium*	0.06 ± 0.04 ^c^	0.47 ± 0.11 ^a^	0.18 ± 0.09 ^b^	↑ *	↓ *

^1^ Values are expressed as the mean ± SD (n = 6). Data in the same column with different superscripts were significantly different at the level of *p* < 0.05. ^2^ ↑ or ↓ indicates the increase or decrease in the relative abundance of gut flora; * indicates significant differences at the level of *p* < 0.05.

## Data Availability

The original contributions presented in the study are included in the article, further inquiries can be directed to the corresponding author.
